# Development and validation of the interpersonal communication assessment tool for assessing the interpersonal communication skills of public health midwives

**DOI:** 10.1186/s12913-023-09511-7

**Published:** 2023-05-24

**Authors:** S.A.S. Prasanna, H.T.C.S. Abeysena, M.A.A.P. Alagiyawanna

**Affiliations:** 1 Ministry of Health, Colombo, Sri Lanka; 2 Department of Community Medicine, Faculty of Medicine, University of Kelaniya, Gampaha, Sri Lanka; 3 Health Promotion Bureau, Ministry of Health, Colombo, Sri Lanka

## Abstract

**Background:**

Interpersonal Communication Skills (IPCS) are one of the core clinical skills that should be developed by the Public Health Midwives (PHMs), who are grass-root level public healthcare providers in primary healthcare settings in Sri Lanka. This study aimed to develop and validate the Interpersonal Communication Assessment Tool (IPCAT), an observational rating scale, to assess the IPCS of PHMs.

**Methods:**

Item generation, item reduction, instrument drafting, and development of the tool’s rating guide were made by an expert panel. A cross-sectional study was conducted in five randomly selected Medical Officer of Health (MOH) areas, the smallest public health administrative division in the district of Colombo, Sri Lanka, to identify the factor structure, which is the correlational relationship between a number of variables in the tool. A sample of 164 PHMs was recruited. The data on IPCS were collected by video-recording the provider-client interaction using simulated clients. All recorded videos were rated by a rater using the drafted IPCAT, which included a Likert scale of 1(poor) to 5 (excellent). Exploratory factor analysis was conducted using the Principal Axis Factoring extraction method and the Varimax rotation technique to explore the factors. Three independent raters were used to rate ten randomly selected videos to assess the tool’s internal consistency and inter-rater reliability.

**Results:**

The IPCAT obtained a five-factor model with 22 items, and all five factors explained 65% of the total variance. The resulting factors were “Engaging” (six items on making rapport), “Delivering” (four items on paying respect), “Questioning” (four items on asking questions), “Responding” (four items on empathy), and “Ending” (four items to assess the skills of ending a conversation productively). The internal consistency, Cronbach’s Alpha value, for all five factors was above 0.8, and the inter-rater reliability (ICC) was excellent (0.95).

**Conclusions:**

The Interpersonal Communication Assessment Tool is a valid and reliable tool for assessing the interpersonal communication skills of Public Health Midwives.

**Trial registration:**

Clinical Trial Registry, Sri Lanka. Ref No, SLCTR/2020/006(February 4th,2020)

**Supplementary Information:**

The online version contains supplementary material available at 10.1186/s12913-023-09511-7.

## Background

Interpersonal Communication (IPC) is a face-to-face interaction that shares information, meanings, and feelings among individuals, verbally and non-verbally. Even though we focus more on verbal communication, it has been identified that more than 60% of human communication is done non-verbally [1, 2].

A specific set of verbal and non-verbal skills has been identified as core skills for effective IPC. These sets of skills are called Interpersonal Communication Skills (IPCS). The recommended IPCS are active listening, empathy, responding to verbal and non-verbal cues, types of questioning, summarizing, planning, structuring, reflecting, clarification, adapting, chunking of information, and checking understanding [3]. IPCS empower the communicator to adjust and manage in talking and listening while identifying the issues occurring during the communication process and managing them for fruitful communication. Therefore, health communicators should be equipped with IPCS to conduct effective and efficient IPC with their clients [4, 5].

There are five core competencies in IPCS. They are (1) creating a therapeutically and ethically sound relationship with the client, (2) active listening skills, (3) eliciting and providing information, (4) using effective verbal and non-verbal questioning, and (5) working effectively with other health care members in the team [6]. Non-verbal skills such as eye contact, head nodding, gesture, posture, and showing interest in improving patient satisfaction also play a 5significant role in IPCS. A healthcare worker competent with these IPCS will enhance client satisfaction [7].

Assessment of IPCS is a complex task. As this assessment is difficult, the researcher should have a reliable and valid tool to assess the IPCS. Observational checklists and rating scales are the most commonly used tools in assessing IPCS worldwide. Observational rating scales contain statements to evaluate each IPCS. These statements are called items. One item is used to determine a particular IPCS. According to the characteristic features of the items in the tool, they are grouped into sub-groups or domains called factors. The tool’s factors are arranged systematically according to the subject being assessed. This structure is called the factor structure. Observational rating scales consist of scoring scales and rating guides for the rater to rate the item according to the rater’s opinion. Checklists do not contain that kind of scoring scale or rating guides and only observe whether the observed skills were performed or not. Therefore, observational rating scales provide more reliable results than checklists in assessing IPCS [8].

Sri Lanka has a well-established primary health care system. Public Health Midwives (PHMs) are frontline primary health care workers providing maternal, reproductive, sexual, and child health care at the grass-root level through domiciliary and field clinics. PHMs should have proper IPCS to interact with their clients and families to empower them to establish healthy behaviors [9]. Therefore, IPCS of PHMs should be measured periodically and evaluated, and if needed, they should be provided with proper training on IPCS. For that, there should be a valid tool to assess IPCS of PHMs, which will also be helpful for training purposes. Few tools have been developed and validated to evaluate IPCS of public health staff in other countries but not in Sri Lanka [7, 10, 11]. Therefore, it is a timely requirement to develop and validate a reliable tool for Sri Lankan context to assess the IPCS of PHMs. This study aimed to develop and validate the Interpersonal Communication Assessment Tool (IPCAT), an observational rating scale to assess the IPCS of PHMs.

## Methods

The IPCAT, an observational rating scale to assess the IPCS of PHMs, was developed by exploring its factor structure through a cross-sectional study using the PHMs working in Colombo district, Sri Lanka. This tool development process consisted of 5 steps.

### Step 1: Item generation

The principal investigator (PI) conducted a literature review to generate the item list of the tool. The theoretical explanations, internationally recommended agreements, and statements published by the experts on IPCS were studied [3, 8, 11]. It was identified that doctors, nurses, and midwives use common IPCS when communicating with their clients. Therefore, few studies of communication skill assessment of nurses and midwives were reviewed, and unpublished IPCS tools developed by local agencies were also studied [12–14]. Items for the new tool were extracted by referring to commonly used observational checklists and rating scales developed for doctors and nurses [8, 15]. The tools studied were the Interpersonal Skill Instrument [16], Standardized Grading Tool for the assessment of IPS [17], Health Communication Assessment Tool [18], SEGUE framework to assess IPCS [19], MAAS Global Rating for the doctor-patient interview [20], and Kalamazoo Consensus framework for doctor-patient communication [21]. The extracted items were pooled, and the initial list consisted of 48 items.

### Step 2: Analyzing the content of the items for item reduction

The PI analyzed the content of 48 items, removed the items duplicating the same meaning, and created a list of 32 items. This list was forwarded to the experts for further reduction of items. Item reduction was carried out by a panel of eight experts who represent the field of public health, psychology, and counseling. These experts had expertise in assessing, planning, and conducting training on IPCS for PHMs. The experts were given 32 items list with its evaluation format, which included a rating scale to rate the items according to their importance in assessing IPCS. The rating scale, a five-point Likert scale starting from 1(least important) to 5 (most important), was used to rate the items based on their importance in assessing IPCS. The experts’ ratings on 32 items were assessed, and the average was calculated for each item. Items with scores of less than three were deemed as being of low importance, and those items were deleted (N = 7). The resulting 25-item list was sent to the expert panel for the second round of suggestions. As all 25 items had high mean values, this 25-item list was forwarded for analysis.

### Step 3: Formulate a draft instrument to measure IPCS

An expert panel, including three experts in multidisciplinary fields from Public Health, Mass Communication, and Medical Education, supervised the drafting process of the item list. The extracted items in the list were modified, rewritten, and converted to standard statements using simple language. The experts reviewed the rewritten items critically concerning their comprehensiveness and appropriateness to their original meaning. The expert team ensured the face, content, and consensual validity of all the items for their relevance, appropriateness, and acceptability to assess the IPCS of PHMs in the local context. After reviewing the literature, a linear continuous judgment scale, a 5-point Likert scale, was selected as the scoring scale by the experts. A five-point scale is a simple rating scale that could be easily used to assess IPCS. The five-point scale provides fewer options/responses compared to the 7-point scale. But raters could use these five responses to provide clear, relevant, and quick answers concerning their genuine emotions. And also, the five responses on the 5-point scale contain a neutral standpoint, making the rater understand opposing extremes. Concerning all the plus points, the expert team recommended a 5-point Likert scale, including the responses of 1-poor, 2-fair, 3-good, 4-very good, and 5-excellent as the scoring scale [22]. The PI developed a tailor-made rating guide for raters, which the expert team approved with some corrections (Supplementary Table 1). The drafted IPCAT, scoring system and rating guide were initially drafted in English and translated to Sinhala language using forward-backward translation methods. Since the draft tool consisted of 25 items, the maximum composite score per each individual could be (25x5) 125 marks, and the minimum composite score (25x1) 25 marks. As the scoring scale mark 3 (good skill) and above represent good IPCS, 60% of the IPCS score was set as the cut-off point for “Good IPCS”.

### Step 4: Exploring the factor structure of IPCAT

A descriptive cross-sectional study was conducted from May 2019 to December 2019 in the district of Colombo, Sri Lanka to explore the factors and factor structure of the drafted IPCAT. This study was conducted in the district’s primary health care setting, called the Medical Officer of Health (MOH) area. Study participants were PHMs, who completed six months of service in the same work setting in the selected MOH area.

The minimum sample size (n = 125) was calculated by adding five participants per item (25x5) [23]. Five MOH areas out of 18 MOH areas in Colombo district were randomly selected to fulfill the sample size. There were 164 eligible PHMs from the selected five MOH settings, and all of them were included in the study. A self-administered questionnaire was used to collect the socio-demographic and service-related data of the PHMs. The data collectors were trained on the questionnaires and the study objectives. In real clinic settings, PHMs are very busy and have many practical disturbances in the clinic environment. Assessing the IPCS with real clients in a real clinic setting is tedious and time-consuming, leading to many errors. Real clients with different educational backgrounds and mental states do not match to test all the aspects of IPCS within a short period of data collection. These difficulties can be overcome by using simulated clients who are trained to facilitate the interview by reacting, behaving, showing feelings, asking, and answering questions to explore the IPCS. Therefore, many studies used simulated clients in the skill assessment studies as it was a cost-effective and feasible method to collect data [24, 25]. The current study also used simulated clients to facilitate the interviews with PHMs. Four females were recruited and trained as simulated mothers having a child with complementary feeding (CF) issues and randomly allocated among the PHMs to reduce selection bias. The interviews of PHMs with these simulated clients were video recorded to collect data on the IPCS of PHMs. The data collectors informed the PHMs of the study objectives and data security. The PHMs were assured that the result of their IPCS performance would not affect their currier development and asked to perform freely with the client as they usually make their conversation in clinics. After assuring the data confidentiality, PHMs’ written consent for video recording was obtained.

It was identified that the Hawthorn effect is a limitation in the data collection process that leads to bias [26]. Therefore, the following preparations were taken to reduce the effect. The PHMs were not given prior notice of the recording date and time to prevent the over preparation for recording. The video recording team was advised to arrange the recording equipment inside the clinic room before commencing the interviews. The data collectors were advised not to be inside the consultation room during the discussion. This was to maintain a comfortable environment for the PHMs where they did not feel they were being observed.

The PI viewed the recorded 164 videos and rated them using the drafted IPCAT following its rating guide. Each item/variable of the IPCAT was given a separate score, and the total score for each PHM was calculated. Data were fed to Statistical Package for Social Science (SPSS) version 20. Exploratory factor analysis was conducted to reduce the number of variables/items. Factors were extracted using principal axis factoring, the common factor analysis approach to extract latent factors. The extracted factors were forwarded to the orthogonal factor rotation method, which assumes that factors are not correlated. This study used the Varimax rotation method to minimize the number of variables that have high loading on each factor, simplifying the interpretation of factors. First, the factorability of the data set was assessed by using the factor correlation matrix and Anti-Image Correlations Matrix. Cronbach’s Alpha values were computed for each factor of the drafted IPCAT to assess the internal consistency.

The instrument’s inter-rater reliability was assessed using three Heath Education Officers (HEOs). These HEOs are the field-level experts who conduct IPC training at the field level in health communication. They also conduct assessments and in-service training for PHMs on IPCS. One-day training was provided for the raters to introduce the drafted IPCAT, its scoring system and the rating guide. After the training, the PI randomly selected ten videos out of previously recorded 164 videos, and these ten videos were used to assess the inter-rater reliability among raters. The three raters rated the ten videos individually, and each video had three ratings from three different raters. Those ratings were analyzed using ANOVA, and intra-class correlation (ICC) was calculated to assess inter-rater reliability.

### Step 5: Finalize the drafted instrument

The drafted IPCAT with latent factor structure was pre-tested among ten raters who work as HEOs. The PI developed a demonstration video depicting a PHM’s interview with a simulated client, a mother having a child with complementary feeding issues, and sent it to the raters through Google Drive. The raters rate the video using the developed tool, the scoring system, and the rating guide. Feedback from each rater was taken by conducting a structured interview to determine the instrument’s clarity. During this structured interview, PI assessed the appropriateness of the wording of the items, availability of unambiguous statements, cleanliness of the items, and the time they had to spend for rating. All the raters mentioned that the wording of the drafted IPCAT was clear, and there was no ambiguous statement. The average time spent rating the tool was 5 to 10 minutes. The developed IPCAT contained 22 items; the maximum total score a participant could achieve was (22x5) 110 marks. “Good skill” is indicated by a score of 3. An individual with “Good skill” for all 22 items of the IPCAT could gain a total of 66 marks, which is 60% of the maximum score (110 marks). Therefore, 60% (66 marks) of the total score of 22 items was taken as the cut-off point of the IPCAT for “Good IPCS.”

## Results

All the participants (n = 164) were females and Sinhalese and had a mean age of 44.7 years (SD = 7.7). The majority of them were in the age category of 30 to 39 years (39.5%), married (86.2%), and passed the Advanced Level examination (60.4%). (Table 1)

**Table 1:** Distribution of the study population by their socio-demographic characteristics (n = 164)

Descriptive characteristics	Frequency	Percentage
**Age category (years)**		
> 60 years 50–59 years 40–49 years 30–39 years < 30years	17 35 31 75 5	8.9 18.4 16.3 39.5 2.6
**Religion**		
Buddhist Catholic Hindu	153 7 4	93.3 4.3 2.4
**Educational level**		
Passed GCE (O/L) Passed GCE (A/L) Above GCE (A/L)	16 99 49	9.7 60.4 29.9
**Marital state**		
Not married Married Divorced Widowed	14 144 2 4	8.4 86.2 1.2 2.4
**Total**	**164**	**100%**

The factor correlation matrix showed more correlations than the accepted level of 0.3 among 25 items, except for two items. These two items (Item No 07 and 10) were removed from the analysis. The Anti-image correlation matrix’s values for the remaining 23 items were well above the accepted level, 0.5. The sampling adequacy for factor analysis was assessed using Bartlett’s Test of Sphericity, which was significant (p < 0.001). The Kaiser-Meyer-Olkin value was 0.89.

One item (Item No 09) with a low factor coefficient value (3.8) was removed from the list and finally formed a list of 22 items. The IPCAT’s modal was identified as a five-factor modal with a range of Eigenvalue from 1.2 to 1.8. The result of factor coefficients for individual items is shown in Table 2. The factor correlation matrix showed that all the factors in this five-factor model were positively correlated. (Table 3)

**Table 2:** The factor coefficients of individual items after Promax rotation in PAF of drafted IPCAT

No	Item	Factors				
		1	2	3	4	5
**19**	Summarized what was discussed.	**.938**				
**17**	Assessed client’s understanding of the problem	**.912**				
**20**	Acknowledged the client and closed the interview.	**.863**				
**18**	Provided time for the client to ask questions.	**.571**				
**13**	Posture and gesture of the provider showed care and concern.		**.946**			
**03**	Conducted small talk and creation of a friendly environment.		**.908**			
**14**	Maintained eye contact appropriately when talking with the client.		**.757**			
**16**	Used facial expressions.		**.719**			
**02**	Introduced himself or herself.		**.491**			
**01**	Greeted warmly and showed interest.		**.410**			
**11**	Elicited relevant client’s beliefs, concerns and expectations	-	-	-	-	-
**21**	Clarified details as necessary with more specific closed ended questions.			**.967**		
**25**	Moved effectively to additional questions			**.931**		
**09**	Open ended vs close ended questions used during the interview.			**.718**		
**08**	Clarity of the questions asked.			**.444**		
**15**	Used empathy to build relationship with the client.				**.771**	
**12**	Responded explicitly to the client statements about ideas, feelings, and values.				**.571**	
**24**	Listened effectively to client’s responses.				**.543**	
**22**	Did not interrupt the client when the client was talking.				**.487**	
**04**	Used appropriate vocal tone and volume, pace for situations.					**.952**
**06**	Explained using words/ terms that are easy for the patient to understand.					**.688**
**23**	Used short sentences instead of long sentences					**.620**
**05**	Used words that show care and concern throughout the interview.					**.616**

**Table 3:** The Factor Correlation Metrix of IPCAT

Factor	1	2	3	4	5
**1**	1.0	0.61	0.53	0.08	0.64
**2**	0.61	1.0	0.58	0.33	0.72
**3**	0.53	0.58	1.0	0.32	0.55
**4**	0.08	0.33	0.32	1.0	0.36
**5**	0.64	0.72	0.55	0.36	1.0

The factors were labeled according to the characteristic features of their items and the theory behind them. The labeled five factors are Factor 1-Ending (Included Item No 17, 18, 19, 20), Factor 2-Engaging (Included Item No 1, 2, 3, 13, 14, 16), Factor 3-Questioning (Included Item No 8, 9, 21, 25), Factor 4-Responding (Included Item No 12, 15, 22, 24) and Factor 5-Delivering (Included Item No 4, 5, 6, 23). This five-factor model with 22 items explained 65.5% of the total variance contributing from factors 1 to 5 by 43.5%, 8.9%, 5.8%, 4.2%, and 2.9%, respectively. As the developed IPCAT should have a rater-friendly format that the raters could use easily, the order of these five factors was rearranged systematically, and the items within each factor were also rearranged. The re-formatted factor structure and its items under each factor are shown in Table 4.

**Table 4:** The re-formatted factor structure and assigned items for each factor

Factor	Items
**1** **Engaging**	**1**- Greeted warmly and showed interest. **2**- Introduced himself or herself. **3**- Posture and gesture of the provider showed care and concern. **4**- Maintained eye contact appropriately when talking with the client. **5**- Used facial expressions. **6**- Conducted small-talk and the creation of a friendly environment.
**2** **Delivering**	**1**- Used appropriate vocal tone and volume, pace for situations. **2**- Used words that show care and concern throughout the interview. **3**- Explained using words/ terms that are easy to understand. **4**- Used short sentences instead of long sentences.
**3** **Questioning**	**1**- Clarity of the questions asked. **2**- Open-ended vs. close-ended questions used during the interview. **3**- Clarified details as necessary with more specific closed-ended questions. **4**- Moved effectively to additional questions
**4** **Responding**	**1**- Used empathy to build a relationship with the client. **2**- Did not interrupt the client when the client was talking. **3**- Listened effectively to the client’s responses. **4**- Responded explicitly to the client’s statements about ideas, feelings, and values.
**5** **Ending**	**1**- Summarized what was discussed. **2**- Assessed client’s understanding of the problem. **3**- Provided time for the client to ask questions. **4**- Acknowledged the client and closed the interview.

All factors’ Cronbach’s Alpha values were well above the accepted level of Nunnally’s criterion 0.7, implying a satisfactory internal consistency [27]. (Table 5) Inter-rater reliability of the IPCAT was excellent (ICC 0.95; 95% CI 0.87–0.98) and above the accepted value of 0.7. Therefore, the rating difference among the raters was not statistically significant.

**Table 5:** Cronbach’s Alpha reliability of IPCAT main domain scores

Main domains	Cronbach’s Alpha	95% CI		Number of items	p value
		Lower bound	Upper bound		
**Engaging**	0.88	0.79	0.89	6	< 0.001
**Delivering**	0.86	0.76	0.88	4	< 0.001
**Questioning**	0.86	0.65	0.88	3	< 0.001
**Responding**	0.82	0.77	0.86	3	< 0.001
**Closing**	0.86	0.79	0.89	4	< 0.001

## Discussion

The IPCAT, an observational rating scale, contains a five-factor model with 22 items. These five factors of the tool explained 65% of the total variance. The resulting factors were “Engaging” (six items on making rapport), “Delivering” (four items on paying respect), “Questioning” (four items on asking questions), “Responding” (four items on empathy), and “Ending” (four items to assess the skills of ending a conversation productively). All the factors in the IPCAT were positively correlated with each other. According to the 5-point scoring scale, the maximum total score that a participant could achieve was (22 items x 5)110 marks. “Good IPCS skill” is indicated by a score of 3. An individual who had “Good IPCS” for all 22 items could gain a total of 66 marks, which is 60% of the maximum score (110 marks). Therefore, 60% (66 marks) of the total score of 22 items was taken as the cut-off point for “Good IPCS”. The participants who got a total score of less than 60% were categorized as “Poor IPCS”.

In this study, the judgmental validity of the IPCAT was assessed. During the tool development, several measures were taken by referring experts to ensure the face, content, and consensual validity. The instrument’s reliability was calculated by assessing internal consistency and inter-rater reliability. The IPCT demonstrated an acceptable validity with excellent reliability.

A similar exploratory factor analysis method was used by Klakovich, who found a three-factor solution with 23 items, and the factors accounted for 60% of its total variance [11]. The same method was used to explore the factor structure of physicians’ communication skills assessment tool and revealed a three-factor model, which explained 60.5% of the total variance [28].

The items of the IPCAT are relatively similar to the items in the Kalamazoo checklist, commonly used to assess doctors’ IPCS. The Kalamazoo tool consists of seven factors. The 5th factor of the IPCAT tool represents the 6th and 7th factors of the Kalamazoo checklist. Factor-3 (Questioning) in the IPCAT and Domain-3 (Gather information) in the Kalamazoo checklist contain similar items [21]. The factor structure of the IPCAT is very similar to the SEGUE framework, an observational checklist widely used in assessing skills in medical communication. IPCAT and SEGUE are similar in the number of factors and the basic theory behind them but not the number of items [13, 19]. A modified version of The Health Communication Assessment Tool for nurses included 17 items under four domains. Factor − 3 of that tool is compatible with factor − 1 of the IPCAT. And they share two similar items, body Language and small talk, which are more important in building a friendly environment with the client before discussing the subject matter. It was identified that a factor named “Empathy” in some tools to assess the therapeutic relationship with clients, but in the IPCAT, empathy is recognized as an individual item and grouped under Factor-4, “Responding” [18, 20].

The item named “used short sentences” under factor-2 of the IPCAT is a newly added item not identified from other tools. This newly added item provides an advantage in assessing the provider’s speech clarity and the client’s understanding of what is talked about. The arrangement of the factor structure and the factor order of the IPCAT is systematic, and it follows the basic steps of the provider-client discussion from beginning to end. As the IPCAT’s factor order is compatible with the routine discussion’s steps, the rating process is more straightforward than other tools and would facilitate the rating process [13, 16, 18, 20].

The basic steps of the tool development process of the IPCAT were similar to the study conducted by Tromp, which was an observer rating scale to assess the communication skills and professional behavior of foreign medical students [29]. Researchers in other fields followed similar steps to the current study when developing their new instruments [30, 31]. Items generation in the present study was done by only conducting a literature search, but some other studies used a more specific item generation method by combining the literature review with critical incident scenarios of the target population [11]. In another study, the items were developed based on literature reviewing and interviewing the target ordinance by analyzing their perspectives [10]. The above methods give more specific items reflecting the target population than the current study method. Items of drafted IPCAT were reduced by a non-statistical straightforward process using experts’ ratings. The PI personally visited the expert panel members for the content analysis and explained the study objectives. But, in other studies, the content analysis was conducted using e-mail-based modified Delphi technics, a widely used method for reducing the item pool when the experts are not physically met [31, 32].

It is recommended to adopt a minimum of five response choices for a rating scale to have a productive outcome [33]. Therefore, the IPCAT included a scoring scale with five response choices, enabling the raters to choose appropriate responses comparing their emotions. A uniform response choice was adapted to all 22 items in the IPCAT, similar to a tool developed in another study to assess doctors’ IPCS [22]. The current tool’s selected rating scale with five responses provides more spectrum to examine the IPCS of participants than the scales used in the Kalamazoo consensus and SEGUE framework to assess doctors’ communication skills [8]. In comparing the IPCAT’s rating guide to the SEGUE, the IPCAT’s guide is more straightforward, item-specific, and simple, which provides a rater-friendly format than the SEGU guide, which is more complex [19].

Compared to other studies, the current study has followed similar procedures in data collection, video recording the interviews, and rating the recorded videos, commonly used low-cost and feasible methods for assessing IPCS worldwide [18, 34]. The Cronbach’s Alpha, which represents the internal consistency reliability, varied from 0.82 to 0.88 among the five domains of the IPCAT, indicating the instrument’s acceptable reliability. Internationally validated observational rating scales used to assess doctors’ and nurses’ IPCS also resulted in satisfactory alpha values for all factors above 0.8, as in the current study [11, 18, 28]. The inter-rater reliability assessed by calculating ICC was excellent (0. 95) in the IPCAT, and it was compatible with the ICC value of the Health Communication Assessment Tool (0.99) and the Common Ground Tool [18, 34]. The Kalamazoo Communication Skills Assessment tool had an ICC range from 0.07 to 0.58 and was a low and moderate value compared to the IPCAT [35]. Therefore, the reliability findings of the IPCAT were compatible with most studies carried out in the communication skill assessment studies.

The Hawthorne effect is a form of reactivity in which subjects improve their behavior when they are aware of being observed. In the current study, the PHMs were informed and aware of the recording of their performance, which masked the PHMs’ actual performance. Information bias during the data collection due to the Hawthorne effect is identified as a limitation in the current study [26, 36]. Sri Lanka is a multicultural country; the main languages spoken are Sinhala, Tamil, and English. The developed IPCAT was validated for the community who use the Sinhala language. Therefore, this validated tool cannot be used to assess IPCS by interacting with clients from different ethnic backgrounds who use different languages, which is a limitation of the IPCAT.

The IPCAT was developed referring to IPCS assessing tools commonly used worldwide and using PI’s practical experiences in IPC. PI is a medical doctor involved in IPC in the preventive health sector for 15 years and has a working experience as a television anchor who has conducted one-to-one interviews for more than 20 years. The IPCAT includes factors that directly assess essential components of IPCS that PHMs should be equipped with when interacting with their clients for productive health communication. Compared to other commonly used assessment tools, the IPCAT tool has a user-friendly format that covers all the essential skills related to IPC. Therefore, IPCAT is a simple tool that can be used in assessing IPCS, and the overall score of the IPCAT for a person is an excellent estimate to evaluate their level of IPCS.

## Conclusions

The IPCAT is identified as a valid and reliable instrument to measure the IPCS of PHMs, and it demonstrated acceptable validity with excellent reliability. Since the IPCAT is a tool with a rater-friendly format, it could be quickly introduced to the public health field to assess the IPCS of PHMs. The IPCAT can also be used to determine staff training needs before planning in-service training and can be used as a teaching, monitoring, and evaluation tool.

## Electronic Supplementary Material

Below is the link to the electronic supplementary material

Supplementary Material 1

## Data Availability

The datasets used and/or analyzed during the current study are available from the corresponding author upon reasonable request.
