# Supplementary material for: Development and validation of the interpersonal communication assessment tool for assessing the interpersonal communication skills of public health midwives
Source: BMC Health Serv Res. 2023 May 24;23:539. doi: 10.1186/s12913-023-09511-7 (PMC10210319; doi:10.1186/s12913-023-09511-7)
Supplement: Supplementary file 1 — Supplementary Material 1 [file 12913_2023_9511_MOESM1_ESM.docx]

**Supplementary Table 1**

**The scoring guide developed for the - observational rating scale – IPCAT**

| **Item 1 -** **Greeted warmly and showed interest**. | | |
| --- | --- | --- |
| **Response** | **Guide for rating the performance** | **Marks** |
| Poor | Did not greet and show interest. | 1 |
| Fair | Did not greet but asked to sit by showing the chair. | 2 |
| Good | Greeted with a smile and asked to sit verbally. | 3 |
| Very Good | Greeted with a smile, asked to sit verbally, and showed interest. | 4 |
| Excellent | Greeted with a smile, ask to sit in a friendly manner, show interest and introduce him/ herself. | 5 |
| **Item 2 -** **Introduced himself or herself** | | |
| **Response** | **Guide for rating the performance** | **Marks** |
| Poor | Did not introduce her/himself. | 1 |
| Fair | Introduced her/himself only by his/her position. | 2 |
| Good | Introduced her/himself and by position as well. | 3 |
| Very Good | Introduced her/himself, his/ her position, and his/her workplace. | 4 |
| Excellent | Introduced her/himself, position, workplace and his/her professionality or qualification to conduct such discussions. | 5 |

| **Item 3 - Posture and Gesture of the provider showed care and concern** | | |
| --- | --- | --- |
| **Response** | **Guide for rating the performance** | **Marks** |
| Poor | Not showed appropriate hand and the body movements. Not sitting, facing the client and posture in a manner showing no respect to the client | 1 |
| Fair | Had hand movements but did not express the idea what was being talked. Sitting facing the client and showed open posture that depict “ready to listen.” | 2 |
| Good | Had appropriate body and hand movements to express the idea what was being talked. Leans towards the client slightly with open posture | 3 |
| Very Good | Effectively used body and hand movements to grab the client attention. Lean towards the client to show concern | 4 |
| Excellent | Excellent use of gesture to emphasize the feeling ideas to client throughout the discussions. Leans towards the client shows interest, care and concern | 5 |
| Excellent | Had appropriate eye contact affirmative head nodding and proper vocal responses throughout the interview. | 5 |

**Factor 1 - Engaging**

| **Item 4 - Maintained eye contact appropriately when talking with the client** | | |
| --- | --- | --- |
| **Response** | **Guide for rating the performance** | **Marks** |
| Poor | No eye contact at all, No head nodding | 1 |
| Fair | No appropriate eye contact, head nodding some time only. | 2 |
| Good | Had eye contact, head nodding most of the time. | 3 |
| Very Good | Had eye contact, head nodding and vocal responses most of the time. | 4 |
| Excellent | Had appropriate eye contact affirmative head nodding and proper vocal responses throughout the interview. | 5 |
| **Item 5 - Used facial expressions** | | |
| **Response** | **Guide for rating the performance** | **Marks** |
| Poor | Did not use appropriate facial expressions. | 1 |
| Fair | Used facial expression at times only. | 2 |
| Good | Used appropriate facial expressions most of the time. | 3 |
| Very Good | Used intensified facial expressions according to the ideas and feelings. | 4 |
| Excellent | Client actively engaged in the discussion because of using facial expressions intensified. | 5 |

| **Item 6 -** **Conducted small talk and creating a friendly environment** | | |
| --- | --- | --- |
| **Response** | **Guide for rating the performance** | **Marks** |
| Poor | Did not make small talk. | 1 |
| Fair | Asked “How are you?” without showing any interest. | 2 |
| Good | Asked few words of “Whereabouts” in a friendly manner. | 3 |
| Very Good | Asked few words of “Whereabouts” in a friendly manner providing time and space to talk. | 4 |
| Excellent | Ask few questions of “Whereabouts” in a friendly manner. Provided time and space to talk. Client showed comfortable feeling with the provider. | 5 |

| **Item 1 -** **Used appropriate vocal tone and volume, pace for situations** | | |
| --- | --- | --- |
| **Response** | **Guide for rating the performance** | **Marks** |
| Poor | Weak voice, volume is low. | 1 |
| Fair | Voice not attractive, Average volume. | 2 |
| Good | Good volume, good tone, good pace | 3 |
| Very Good | Good Volume, Good variation of tone and proper pace. Clear pronunciation | 4 |
| Excellent | Uses volume, Tone, and pace appropriately. Clear pronunciation appropriately for the situation. | 5 |

| **Item 2 -** **Used words that show care and concern throughout the interview** | | |
| --- | --- | --- |
| **Response** | **Guide for rating the performance** | **Marks** |
| Poor | Used words not showing respect to the client. | 1 |
| Fair | Used words with some respect. | 2 |
| Good | Used words with moderate respect. | 3 |
| Very Good | Used words with keen respect. | 4 |
| Excellent | Used words with intense respect. | 5 |

| **Item 3 - Explained using words/ terms that are easy for the patient to understand** | | |
| --- | --- | --- |
| **Response** | **Guide for rating the performance** | **Marks** |
| Poor | Used more words/Terms that couldn’t be understood. | 1 |
| Fair | Used some words/Terms that couldn’t be understood. | 2 |
| Good | Used words/Terms that could be understood. | 3 |
| Very Good | Used words/Terms that could be understood clearly. | 4 |
| Excellent | Cleverly used words/terms that could be easily understood | 5 |
| **Item 4 - Used short sentences instead of long sentences** | | |
| **Response** | **Guide for rating the performance** | **Marks** |
| Poor | Asked questions with a collection of long sentences. | 1 |
| Fair | Used long sentences | 2 |
| Good | Used short sentences | 3 |
| Very Good | Used short, simple sentences | 4 |
| Excellent | Used short simple sentences with proper spacing | 5 |

**Factor 2 - Delivering**

| **Item 1 - Clarity of the questions asked** | | |
| --- | --- | --- |
| **Response** | **Guide for rating the performance** | **Marks** |
| Poor | Questions offered are not clear. More questions had to be offered in order to get the answer. | 1 |
| Fair | Client in some way understands the questions those are offered. | 2 |
| Good | Questions those offered were clear to client. | 3 |
| Very Good | Questions those offered were very clear. | 4 |
| Excellent | Questions those offered were very clear. Client realized questions very well and responded appropriately | 5 |

| **Item 2 -** **Open ended vs. close ended questions used during the interview.** | | |
| --- | --- | --- |
| **Response** | **Guide for rating the performance** | **Marks** |
| Poor | Asked only closed ended questions. | 1 |
| Fair | Asked more closed ended question but few open-ended questions. | 2 |
| Good | Asked more open-ended question and asked closed ended questions if need. | 3 |
| Very Good | Asked more open-ended question and clarifies details with specific closed ended questions. | 4 |
| Excellent | Began with open ended questions and continued with more open-ended questions and moved effectively to leading closed ended question. | 5 |
| **Item 3 - Clarified details as necessary with more specific closed ended questions** | | |
| **Response** | **Guide for rating the performance** | **Marks** |
| Poor | Only a closed ended questions were asked. | 1 |
| Fair | At times, closed ended questions were asked for what is needed to explore (inquire) | 2 |
| Good | Closed ended questions were asked accordingly at what is needed to explore (inquire) | 3 |
| Very Good | Closed ended questions were asked accordingly at appropriate moments. for what is needed to explore (inquire) | 4 |
| Excellent | Offers several closed ended questions until the relevant answer comes for what is needed to explore (inquire) | 5 |

**Factor 3 - Questioning**

| **Item 4 - Moved effectively to additional questions** | | |
| --- | --- | --- |
| **Response** | **Guide for rating the performance** | **Marks** |
| Poor | Did not offer any additional questions | 1 |
| Fair | Additional questions were offered at times. | 2 |
| Good | To receive necessary extra information more questions were offered | 3 |
| Very Good | To prolong the discussion additional questions were offered in order to receive additional information. | 4 |
| Excellent | Encouraging the client, the discussion was prolonged by offering additional questions | 5 |

| **Item 1 -** **Used empathy to build relationship with the client** | | |
| --- | --- | --- |
| **Response** | **Guide for rating the performance** | **Marks** |
| Poor | Not showed empathy at all. | 1 |
| Fair | Tried to show empathy but couldn’t understand the client situation. | 2 |
| Good | Showed empathy appropriately by getting the client situation. | 3 |
| Very Good | Effectively got down to the patient level and got a clear understanding of the patient situation. | 4 |
| Excellent | Showed empathy properly and client showed satisfaction | 5 |

| **Item 2 - Did not interrupt the client when the client was talking** | | |
| --- | --- | --- |
| **Response** | **Guide for rating the performance** | **Marks** |
| Poor | Always interrupts when the client is talking. | 1 |
| Fair | Interrupts sometimes when the client is talking. | 2 |
| Good | Did not interrupt when the client is talking. | 3 |
| Very Good | Allowed the client to complete opening statement and did not interrupt throughout the discussion. | 4 |
| Excellent | Not interrupting at all. listens effectively and allows adequate time to talk. | 5 |

**Factor 4 - Responding**

| **Item 3 - Listened effectively to client’s responses.** | | |
| --- | --- | --- |
| **Response** | **Guide for rating the performance** | **Marks** |
| Poor | Did not listening | 1 |
| Fair | Pretending to listen at times | 2 |
| Good | Listening was good | 3 |
| Very Good | Activity engaged in listening to the client | 4 |
| Excellent | Listening was demonstrated by using appropriate verbal and nonverbal expressions. | 5 |
| **Item 4 - Responded explicitly to the client statements about ideas, feelings, and values** | | |
| **Response** | **Guide for rating the performance** | **Marks** |
| Poor | Did not respond | 1 |
| Fair | Responded at times to what the client says | 2 |
| Good | Responded well, according to what the client says | 3 |
| Very Good | Realizes the level of mentality of the client and responded to it | 4 |
| Excellent | Responded by encouraging the client with appropriate verbal and nonverbal expressions. | 5 |

| **Item 1 -** **Summarized what was discussed** | | |
| --- | --- | --- |
| **Response** | **Guide for rating the performance** | **Marks** |
| Poor | Did not summarize what was discussed | 1 |
| Fair | Only several parts of the discussion were summarized | 2 |
| Good | Summarized all necessary points | 3 |
| Very Good | Points were summarized in a manner that the client can remember | 4 |
| Excellent | Points were summarized very simply, clearly and in a logical manner where the client could memorise or remember. | 5 |

**Factor 5 - Ending**

| **Item 2 -** **Assessed client’s understanding of the problem** | | |
| --- | --- | --- |
| **Response** | **Guide for rating the performance** | **Marks** |
| Poor | Did not give any time to ask questions. | 1 |
| Fair | Only ask whether the client understood the discussion and not encouraged the client to ask questions. | 2 |
| Good | Asked the client to forward any questions for clarification what was discussed. | 3 |
| Very Good | Allowed the client to ask questions for further clarification and encouraged the client. | 4 |
| Excellent | Allowed the client to asked questions. Asked the client to say what he/she understood from the session and letting him/her to clarity what was understood. | 5 |
| **Item 3 - Provided time for the client to ask questions** | | |
| **Response** | **Guide for rating the performance** | **Marks** |
| Poor | No opportunity was given to client to ask questions. | 1 |
| Fair | Allowed to ask questions only at some instances. | 2 |
| Good | Allowed to ask questions in many instances | 3 |
| Very Good | Allowed at all appropriate moments to ask questions | 4 |
| Excellent | Allowed to ask questions at any moment encouraging the client for that. | 5 |
| **Item 4 - Acknowledged the client and closed the interview** | | |
| **Response** | **Guide for rating the performance** | **Marks** |
| Poor | Pausing the discussion at once without a warm send off. | 1 |
| Fair | Sent off without a pleasing ending. | 2 |
| Good | Thanked the client for coming and had a pleasant send off | 3 |
| Very Good | Thanked the client and asked to visit again if needed. had a pleasant send off | 4 |
| Excellent | Friendly affective touch, discussed future visits and the client happily left. | 5 |
